# A236 THE EFFECTIVENESS AND SAFETY OF DUAL ADVANCED THERAPIES IN PATIENTS WITH INFLAMMATORY BOWEL DISEASE: A CASE-SERIES

**DOI:** 10.1093/jcag/gwad061.236

**Published:** 2024-02-14

**Authors:** N C Howarth, A Wilson, T Boyd, S Rohekar, P Basharat

**Affiliations:** Western University, London, ON, Canada; Western University, London, ON, Canada; Western University, London, ON, Canada; Western University, London, ON, Canada; Western University, London, ON, Canada

## Abstract

**Background:**

Patients with inflammatory bowel disease (IBD) refractory to medical therapy and patients with IBD and concurrent extra-intestinal manifestations (EIMs) pose a challenge within gastroenterology. The small number of approved agents for IBD limits therapeutic options for this population. Utilizing a combination of newer biologic therapies with favourable safety profiles may provide a safe and efficacious option for treating patients with refractory disease or patients with well-controlled IBD but uncontrolled EIMs.

**Aims:**

The aim of our study was to assess the safety and efficacy of dual advanced therapies for patients with IBD.

**Methods:**

Patients at London Health Sciences Centre and St. Joseph’s Healthcare Centre on dual advanced therapies for inflammatory bowel disease were retrospectively assessed for a minimum period of 12 months. Major bacterial infection, adverse events, endoscopic, histologic, and clinical remission of IBD, and remission of EIMs of IBD were assessed.

**Results:**

Five patients with Crohn’s disease receiving dual advanced therapies were identified. Stricturing Crohn’s disease was the predominant phenotype in 80% of the patients. One patient had perianal fistulizing disease. Dual therapy was initiated for concurrent management of EIMs in 80% of patients (enteropathic arthritis in 2 patients, spondyloarthritis in 2 patients, rheumatoid arthritis in 1 patient) and for refractory IBD in one patient. The majority of patients were on a combination of a tumour-necrosis-factor inhibitor and vedolizumab. Other advanced therapies included JAK inhibitors and ustekinumab. The patients were predominantly female (80%) and age at diagnosis was ampersand:003C50 years old in 80% of patients. No adverse events or major bacterial infections were observed during dual therapy. At the end of follow-up, all 5 patients were in clinical remission of their IBD and 4 patients demonstrated endoscopic remission. Only 60% of patients had remission of their EIMs within the follow-up period.

**Conclusions:**

Our findings suggest that dual advanced therapies are safe and may represent a long-term treatment option for complicated patients with IBD.

**Funding Agencies:**

None

